# Transient Three-Dimensional Flow Field Measurements by Means of 3D µPTV in Drying Poly(Vinyl Acetate)-Methanol Thin Films Subject to Short-Scale Marangoni Instabilities

**DOI:** 10.3390/polym13081223

**Published:** 2021-04-10

**Authors:** Max Tönsmann, Philip Scharfer, Wilhelm Schabel

**Affiliations:** Karlsruhe Institute of Technology (KIT), Thin Film Technology (TFT), Kaiserstraße 12, 76131 Karlsruhe, Germany; philip.scharfer@kit.edu (P.S.); wilhelm.schabel@kit.edu (W.S.)

**Keywords:** polymer film drying, Marangoni number, particle tracking velocimetry, flow field, convective instability

## Abstract

Convective Marangoni instabilities in drying polymer films may induce surface deformations, which persist in the dry film, deteriorating product performance. While theoretic stability analyses are abundantly available, experimental data are scarce. We report transient three-dimensional flow field measurements in thin poly(vinyl acetate)-methanol films, drying under ambient conditions with several films exhibiting short-scale Marangoni convection cells. An initial assessment of the upper limit of thermal and solutal Marangoni numbers reveals that the solutal effect is likely to be the dominant cause for the observed instabilities.

## 1. Introduction

Thin functional polymer films are a vital component in many products, such as displays, battery separators, or membranes for fuel cells. Commonly, film thickness variations should not exceed 1% to ensure product functionality [1]. Since polymer films are manufactured from solvent solution through coating and subsequent film drying, convective instabilities in the liquid film potentially induce surface deformations, which persist in the dry polymer film [2]. Accordingly, knowledge regarding the influence of drying conditions on convective instabilities is required.

Convective instabilities in thin liquid films are predominantly driven by surface-tension gradient induced Marangoni convection. Bénard reported regular patterns of vertical convection cells in non-volatile pure liquid films heated from below more than 100 years ago. The lateral length scale of the convection cells was similar to the film height [3,4]. Accordingly, such convection cells are termed short-scale Bénard–Marangoni convection. Among the first to suggest surface-tension gradients as a driving force of such convection cells was Pearson, who performed a theoretic linear stability analysis, assuming a non-volatile pure liquid film with a rigid free surface heated from below [5]. The results revealed a dimensionless number, later to be named Marangoni number $Ma_{T}$, as a measure to estimate whether a film is convectively stable or exhibits short-scale Bénard–Marangoni convection cells. Pearson’s analysis revealed a lower limit for $Ma_{T}\geq 80$, below which no Bénard–Marangoni convection cells may occur in a film with constant temperature at the bottom. This threshold value for cellular instability is known as the critical Marangoni number $Ma_{T,crit}.$ It is not a constant value, but depends on the boundary conditions. For a constant heat-flux boundary condition at the bottom of the film, Pearson’s analysis revealed $Ma_{T}\geq 48$ and for both, lower boundary conditions $Ma_{T,crit}$ increased with increasing upper Biot number. The author also implied that buoyancy-driven convective instabilities are relevant only for a film thickness h>1 mm, which still appears to be consensus in the present. In order to assess whether buoyancy or surface tension gradients are the driving force of convective instabilities, it is established to calculate the dimensionless Bond number Bo, where Bo≫1 implies the dominance of buoyancy and Bo≪1 indicates surface tension driven Marangoni convection [6,7,8].

Experimental findings from evaporating thin solvent films were reported by Zhang et al. [9,10]. All investigated films exhibited Bénard–Marangoni convection cells even when cooled from the bottom, which should be stable according to Pearson’s and many following theoretical analyses. Several comprehensive reviews provide more information on thermally induced Marangoni convection [8,11,12,13].

An additional level of complexity arises when liquid mixtures or solutions are considered. The surface tension then becomes dependent not only on temperature, but also on concentration [14]. Regarding polymer film drying, the decreasing solvent content during drying leads to a strong viscosity increase dampening any flow. Several authors explicitly state the stabilizing effect of viscosity increase during polymer film drying [15,16,17,18]. In addition, the mass diffusion coefficient strongly decreases, especially for low solvent concentrations [19]. This is important since solvent diffusion mitigates concentration gradients and therefore solutally induced Marangoni convection. It has to be noted that several authors report an additional (in)stability mechanism in ultrathin films with a thickness in the nanometer range (≤100 nm), where inter- and intramolecular forces become relevant, especially for (de)wetting films [20,21,22,23,24,25]. For additional details regarding this topic, the readers are referred to the aforementioned references and [8].

Solutally induced Marangoni convection was investigated theoretically in various configurations [15,26,27,28,29,30]. Available experimental work on convective Marangoni instabilities in drying polymer films appear to be governed primarily by a deliberate use of Marangoni convection as means for self-assembly [18,31,32,33], including the transition between different modes of instability and lateral patterns of convection cells [17,18,34,35,36,37,38,39]. A quantitative assessment of the stability threshold of solutally induced Marangoni convection in drying polymer films based on experimental findings, as reported in [17,18], can be found but appears to be less frequent. Bormashenko et al. reported very regular hexagonal cell patterns in diluted polymer solution films produced by dip coating. Although the authors did not provide viscosity values, the dip-coating process implies a rather low initial viscosity [40,41]. Other authors reported less regular cells with different polygonal cells and large lateral size distributions having a standard deviation in the order of 20 to 50% [17,18,37,39]. In addition, it was found that the lateral cell size increased with increasing initial polymer concentration [32,39] and increasing initial film thickness [17,18,39]. Minarik et al. investigated the impact of different molecular weight (MW) of 2-hydroxyethyl cellulose in aqueous solutions and found that for low molecular weight and narrow distributions fairly regular polygonal cells emerged, whereas for large MW and broad distributions very irregular patterns were found.

When thermal and solutal gradients arise simultaneously, it raises the question whether thermal or solutal effects are the main cause for Marangoni instabilities. Several authors conclude that the solutal effect is dominating [15,29,42].

Experimental data on the stability threshold of Marangoni instabilities in drying polymer films are, however, scarce. Several authors have acknowledged this in the past [39,43,44]. In a recent comprehensive review on multiphase Marangoni convection, Wang et al. explicitly stress the need for new quantitative experiments regarding convective instabilities in drying polymer films [44]. Bassou et al. reported experimental findings of cellular convective instabilities in drying polystyrene-toluene films with an initial thickness of 55 μm–1.5 mm [18]. Calculating the thermal and solutal Marangoni numbers revealed thermal Marangoni numbers below available critical thresholds, whereas the found solutal Marangoni numbers were significantly larger. The authors conclude that this implies the dominance of solutally over thermally driven instabilities. Toussaint et al. investigated drying polyisobutylene–toluene films with an initial wet-film thickness in the range of 0.3 to 14.3 mm, an initial polymer mass fraction of 0 to 15 wt%, and an initial viscosity of 0.55 to 2100 mPa⋅s [17]. They observed Bénard-like convection cells for an initial film thickness $h_{0}\leq 4\;\mathrm{mm}$ and for thicker films they found roll cells persisting even after a solvent-depleted surface layer formed during drying. Toussaint et al. attributed these forms of convective instability to Marangoni effects and buoyancy-driven Rayleigh convection, respectively. In a follow-up publication, Touazi et al. compared the experimental findings to a theoretic stability analysis accounting for thermally induced Marangoni and buoyancy-driven convective instabilities including a realistic viscosity increase [45]. The results were in reasonable agreement with the experimental findings of Toussaint et al., and the found critical thermal Marangoni number $Ma_{T,crit}$ was in the order of $10^{2}$ to $10^{5}$. Subsequently, Doumenc et al. also derived a numeric model accounting for solutally induced Marangoni convection and a realistic viscosity increase [46]. The model revealed critical solutal Marangoni numbers $Ma_{s,crit}$ being also in the order of $10^{2}$ to $10^{5}$, but no experimental validation was provided for the solutal model.

The drying and shrinkage process of polymer film drying is typically regarded as a one-dimensional (1D) process perpendicular to the film plane, accounting for 1D solvent diffusion in the film, the phase equilibrium at the free surface, and 1D solvent transport in the gas phase [19,47,48,49,50,51,52]. The diffusion coefficient as well as the phase equilibrium of polymer solutions commonly exhibit a low concentration dependency for diluted solutions with values close to the properties of the pure solvent [48,49,53]. During polymer film drying of diluted solutions, this results in an initially constant evaporation rate where the solvent transport is solely limited by the mass transport in the gas phase [19,54,55,56,57]. Towards lower solvent concentrations, the diffusion coefficient becomes strongly concentration-dependent, decreasing by several orders of magnitude with decreasing solvent content [48,49,53]. At this stage, polymer film drying becomes limited by the solvent diffusion in the film and the evaporation rate decreases significantly [19,47,48]. The concentration-dependent diffusion coefficient can be derived from the free-volume theory [58], from gravimetric [57,59] or Raman-spectroscopy based experiments [53,60]. In addition, the pure polymers may exhibit glass transition. The glass transition temperature decreases significantly when adding a solvent, which acts as a plasticizer [54,61,62]. When the glass transition temperature of the pure polymer is significantly higher than the drying temperature, it is well known that a drying polymer film may undergo a transition from rubbery to glassy state during drying, when the decreasing solvent content leads to an increase of the glass transition temperature exceeding the drying temperature [48,52,63,64]. Therefore, when the pure polymer’s glass transition temperature is larger than the drying temperature, it is a possibility that a glassy surface layer emerging during drying inhibits further surface tension gradient induced Marangoni instabilities due to the strong viscosity increase associated with glass transition [61,65,66,67]. The glass transition temperature of the pure polymer investigated in this study (poly(vinyl acetate)) is between 29 °C and 33 °C, and it was shown that with addition of 4 wt% methanol the glass transition temperature drops under 15 °C [19], which is below the experimental conditions used in this study. In the case, that the pure polymer’s glass transition temperature is far larger than the drying temperature, it is more likely that a glassy surface layer emerges during drying, inhibiting surface tension gradient induced convective instabilities. The effect of glass transition during drying was recently investigated in our group in poly (methyl methacrylate) ($T_{g}\approx 105\;{}^{\circ}C$) films [64]. In addition, Toussaint et al. reported convective instabilities in drying polyisobutylene–toluene solution films and pointed out that Marangoni instabilities may seize, when a solvent-depleted viscous skin emerges at the surface of the drying films, even when the bulk viscosity is still low. However, the possible effect of glass transition was not mentioned explicitly [17].

In our group, research regarding the experimental investigation of polymer-solution film drying has been conducted in the past. A Raman spectroscopy based measurement technique was developed, giving access to transient vertical concentration profiles in drying films under varying drying conditions [19,60,68]. The concentration- and temperature-dependent mutual mass diffusion coefficient was determined using a sorption balance [57,59] and Raman drying experiments [53,60]. The transient vertical concentration profiles of various polymer solutions could be described accounting for Fickian diffusion and a concentration-dependent diffusion coefficient in the film [19,47,53], whereas other authors found sigmoidal-shaped solvent concentration profiles, which were modeled assuming a viscoelastic contribution to diffusion [51,52,63].

The surface deformation of a poly(vinyl acetate)-methanol film subject to deliberate laterally inhomogeneous drying conditions was examined experimentally in our group (initial wet-film thickness $h_{o}\approx 150\;\mu m$, substrate temperature $T_{substrate}=30\;{}^{\circ}C$, drying at ambient conditions) [42]. The laterally inhomogeneous drying conditions were achieved by using a temperature-controlled metallic substrate with a partial Teflon inlay. A comparison with non-isothermal drying simulations revealed a surface temperature of the film of $T_{surface}\approx 28\;{}^{\circ}C$ above the metallic portion of the substrate and $T_{surface}\approx 20\;{}^{\circ}C$ above the Teflon inlay. In combination with experimentally derived temperature- and concentration-dependent surface tension data, it was further assessed that the solutal effect was the dominant driving force for the surface deformation [42].

In order to further investigate the convective instability causing such surface deformations, we have recently established a microscopic measurement technique based on particle tracking velocimetry and designed for quantitative measurements of the transient three-dimensional flow-field in drying thin films (three-dimensional micro particle tracking velocimetry, 3D-µPTV, see Section 2.2) [69,70].

The aim of this work is the experimental investigation of short-scale convective instabilities in drying poly(vinyl acetate)-methanol films using 3D-µPTV. It will be shown that some films are convectively stable during drying, while others show initial short-scale Bénard–Marangoni convection cells, becoming stable during the constant rate period of drying. We further report velocity distributions in such films and make initial assessments regarding the upper limit of thermal and solutal Marangoni numbers. A rigorous quantitative assessment of the transient Marangoni numbers requires the knowledge of vertical temperature and concentration gradients as well as solution properties. Therefore, this will be addressed in a separate study in the future.

## 2. Materials and Methods

### 2.1. Materials, Solution Preparation, Coating, and Drying

**Figure 1:**
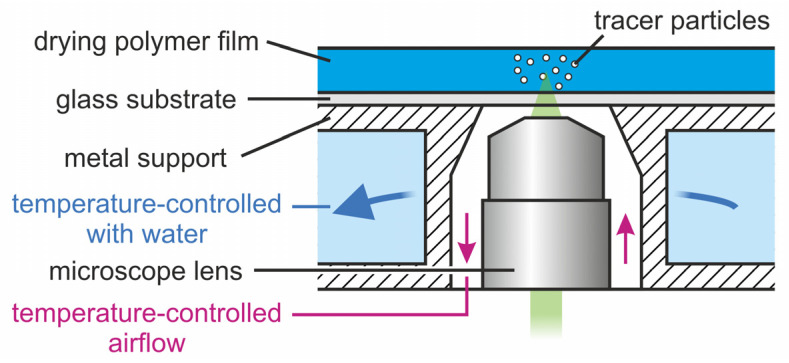
Schematic drawing of the substrate temperature control 3D-µPTV drying experiments.

Binary polymer solutions were prepared from poly(vinyl acetate) (PVAc, Carl Roth, 9154.1) and methanol (MeOH, Carl Roth, 4627.1) by weight with an initial solvent load of $X_{0}=1$, 1.5, and $2\;g_{MeOH}/g_{PVAc}$. The solutions were mixed on a roll mixer at ambient temperature for at least one week. Films were blade coated with custom coaters and coating gaps of $h_{gap}=50$, 100, 150, and 200 μm on microscope-grade borosilicate glass substrates with a thickness of approximately 150 μm. The films had an approximate extent of 5*–*8 cm in coating direction and 2 cm in cross-coating direction, respectively. The glass substrates were mechanically attached on top of a hollow metallic support, temperature-controlled by water thermostats set to $T_{substrate}=20\;{}^{\circ}C$. The metallic support had an opening in the center for optical access to the film from below, which is necessary for transient microscopic flow-field measurements during drying, detailed in Section 2.2. The opening is also temperature controlled from below with a temperature-controlled airflow around the tip of the microscope lenses (see Figure 1). Drying of the films was performed under ambient conditions. In order to mitigate the effect of lab ventilation airflow on drying, the films were covered with a box of approximately 15 cm edge length with an open top.

### 2.2. Transient 3D Flow-Field Measurements

We have recently established a microscopic measurement technique based on particle tracking velocimetry (3D-µPTV), designed to measure the transient three-dimensional flow-field in drying thin films. It is based on microscopic tracking of nanoscale fluorescent tracer particles through a thin glass substrate from below, hence the opening in the temperature-controlled block mentioned in Section 2.1 (Figure 1). A method called “off-focus imaging”, first proposed by Speidel et al., allows for the detection of vertical particle movements by evaluating the diffraction rings, occurring when the tracer particles are not in focus [71]. The diffraction ring diameter increases with increasing vertical distance to the focal plane. This allows for 3D measurements with a single camera. However, with increasing ring size, the signal-to-noise ratio deteriorates, which limits the vertical field-of-view of a single camera. Therefore, we have combined this approach with multifocal microscopy: the fluorescent signal of tracer particles is distributed with beam splitters to up to five cameras, supplemented with motorized lens systems that allow for vertical focal plane adjustment for each camera individually. Details of the physical setup have been published elsewhere [69].

In this work, we use three cameras with a resolution of 640 px×540 px (4×4 pixel binning) and a recording speed of 10*–*30 fps as well as a microscope lens with magnification 60× (Plan Apo λ, air immersion, Nikon, Tokyo, Japan). This results in a field-of-view of approximately 250 μm lateral diameter and the entire height of the film under investigation (maximum initial wet-film thickness $h_{0}\approx 100\;\mu m$). The uncompressed raw video data size from a single experiment lasting approximately 5–10 min is in the order of 10 GB. For this amount of data, manual ring detection is virtually impossible. Therefore, a ring-detection algorithm detailed in [69] was utilized. The calibration routine to match the detected diffraction-ring diameters to vertical particle positions, as well as trajectory stitching and velocity calculation, has been published in [70]. The calibration of vertical tracer-particle positions is dependent on the refractive index of the film under investigation, which changes with solvent concentration (see Section 2.3). Velocity distributions are evaluated with the mean refractive index derived from initial solvent load and dry polymer. An assessment of the experimental errors is provided in Appendix C.

### 2.3. Material Properties

Several material properties of the used PVAc-methanol solutions are required for experiment evaluation, as well as for the assessment of the upper limit of Marangoni numbers: These are the density ρ and the refractive index n for 3D-µPTV experiments, as well as the surface tension σ, dynamic viscosity η, thermal diffusivity a and mutual diffusion coefficient D for thermal and solutal Marangoni number estimation.

**Figure 2:**
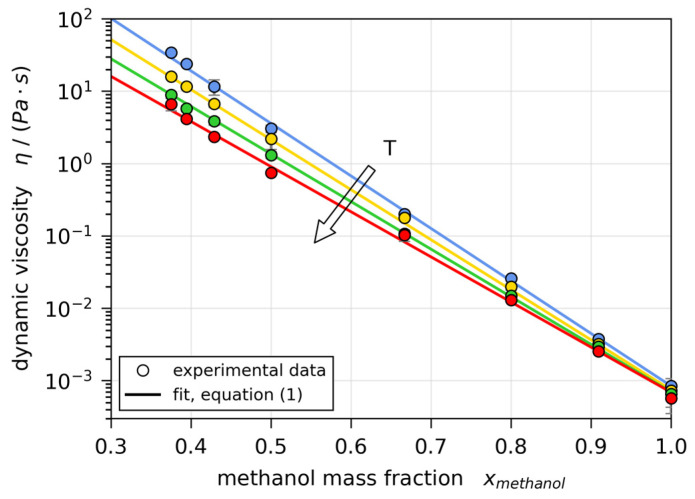
Concentration- and temperature-dependent dynamic viscosity η of poly(vinyl acetate)-methanol solutions for T=10, 20, 30, and 40 °C.

The viscosity, known to strongly increase with decreasing solvent content, was measured using a rotary type rheometer (MCR 101, Anton Paar, Graz, Austria) with a shear rate between 0.1 and $1000\;s^{-1}$. The experimental data show no shear rate dependency, indicating Newtonian behavior of the coating solution. The data were fitted using the relation

(1) $$\eta =a\times exp\left\{\frac{b}{T}+\left(c_{0}+T\times c_{1}\right)\times x_{MeOH}\right\}$$

with η being the dynamic viscosity, T the temperature in K and a, b, $c_{0}$, and $c_{1}$ fit parameters (Figure 2). The relation was originally reported in [72], but we have added the linear dependency on temperature of parameter c to better represent our experimental data.

The remaining material properties and their respective sources are provided in Appendix A (Table A1, Table A2, Table A3 and Table A4).

### 2.4. Marangoni Number Assessment

It will be shown that the largest initial wet film thickness of the experiments presented in this work was $h_{0,max}\approx 100\;\mu m$ (Section 3.2). The assessment of Pearson regarding the driving mechanism implies that surface tension gradient induced Marangoni convection should be dominant, whereas buoyancy-driven instabilities should be negligible. In line with other available publications, the upper limit of the dimensionless Bond number

(2) $$Bo_{max}=\frac{\rho_{max}\times g\times h_{max}^{2}\;}{\sigma_{min}}$$

with $g=9.81\;m/s^{2}$, was calculated to clarify this.

In order to assess the upper limit of thermal and solutal Marangoni numbers, we consider only the highest possible value by making estimates for all relevant parameters. A more detailed analysis is planned for future work. Accordingly, the highest estimated values in the numerator and the lowest estimated values in the denominator are considered. The upper thermal Marangoni number limit $Ma_{T,max}$ was calculated as

(3) $$Ma_{T,max}=\Delta \sigma {\left(T\right)}_{max}\times \frac{h_{max}\;}{\eta_{min}\times a_{min}},$$

with Δσ being the surface tension difference between the surface and bottom of the film. The upper solutal Marangoni number limit $Ma_{s,max}$ was defined accordingly as

(4) $$Ma_{s,max}=\Delta \sigma {\left(x\right)}_{max}\times \frac{h_{max}}{\eta_{min}\times D_{min}}.$$

The subscript of Ma denotes whether the instability is driven by thermal or solutal effects. These definitions are equivalent to previously published works. Note that the vertical surface tension difference Δσ is occasionally given as $\partial \sigma /\partial R\cdot {\Delta R}_{\mathrm{vertical}}$, with R being either temperature or concentration.

## 3. Results

The results are structured as follows: initially, we report the flow fields in convectively stable and initially unstable drying films measured with 3D-µPTV (Section 3.1). In the following Section 3.2 to Section 3.4, the deduced drying curves, velocity distributions, and lateral convection cell sizes are presented and discussed, respectively. Finally, findings regarding the Marangoni stability threshold as well as initial estimations of the upper limit of thermal and solutal Marangoni number are provided in Section 3.5.

### 3.1. Convective (In)Stability

**Figure 3:**
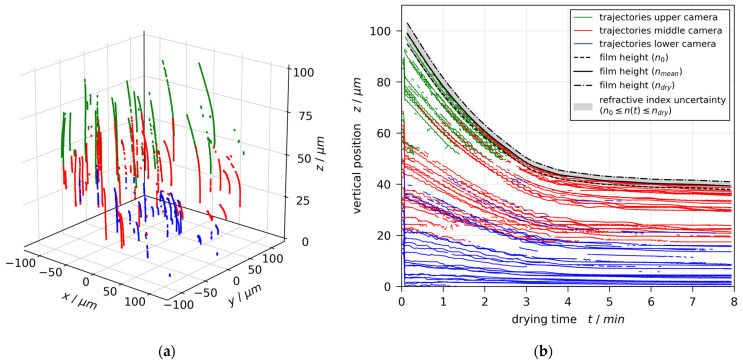
Flow field in a poly(vinyl acetate)-methanol film ($X_{0}=1\;g_{MeOH}/g_{PVAc}$ and $h_{gap}=200\;\mu m$ ), drying at $T_{substrate}=20\;{}^{\circ}C$ and otherwise ambient conditions, measured with 3D-µPTV and evaluated with mean refractive index $n_{mean}$. The flow field is dominated by film shrinkage. (**a**) The 3D tracer particle trajectories. (**b**) Transient vertical tracer particle positions. The extracted drying curves are additionally given for an evaluation with the refractive index of the coating solution $n_{0}$ and the dry polymer $n_{dry}$, respectively (black curves).

The particle trajectories measured with 3D-µPTV in an evaporating film of poly(vinyl acetate)-methanol with initial solvent load $X_{0}=1\;g_{MeOH}/g_{PVAc}$ and coating gap $h_{gap}=200\;\mu m$ are given in Figure 3a. It can be clearly seen that the particle tracks are solely vertical, showing no convective instability. Figure 3b shows the same tracks’ vertical position in the film z over drying time t. The vertical particle movement is directed in negative z-direction, solely following the film shrinkage due to methanol evaporation.

A few 3D-µPTV-related observations have to be discussed: at approximately 5 s, a strong and almost instant decrease of particle positions can be seen. This is due to the fact that the coater is pressed on the substrate during coating, temporarily changing the substrate top position relative to the focal planes of the observing cameras. After coating is complete, no further disturbances of this kind are found. Note as well that the transient particle positions show an almost step-like decrease of z-positions. This is associated with the low camera resolution, utilized to reduce the size of the raw video data. Finally, there are some data gaps observable, e.g., starting at t≥0 s and z≈65 μm. Since µPTV is a discrete measurement technique, information of the flow field can only be extracted where tracer particles can be detected. These gaps depend on the tracer particle distribution in the drying film. Transient gaps in particle trajectories throughout the experiment may also occur when the ring-detection algorithm fails to detect them. Nevertheless, the µPTV results clearly show the characteristics of the flow field. To the best of our knowledge, this is the first reporting of a fully-fledged transient three-dimensional flow field in a thin drying film of micrometer scale.

In order to extract the drying curve, a hull curve accounting for the highest observed particle positions is shown in Figure 3b as black solid line. Since the calibration of µPTV is dependent on the film’s refractive index, which changes during the drying experiment, the evaluation was repeated with the initial refractive index of the coating solution $n_{0}$ (black dotted line) as well as the refractive index of dry poly(vinyl acetate) $n_{dry}$ (black dash-dotted line).

**Figure 4:**
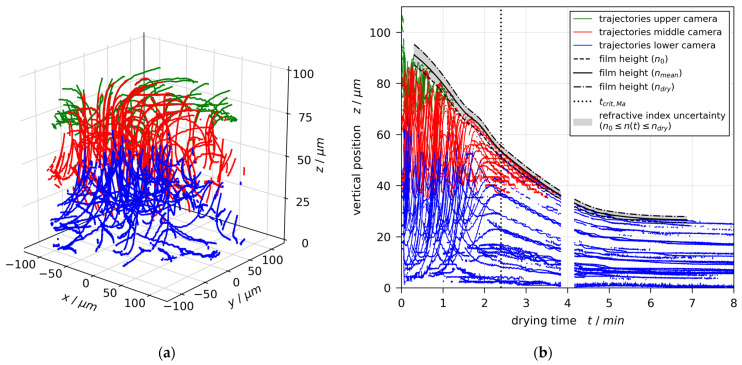
Flow field in a poly(vinyl acetate)-methanol film ($X_{0}=1.5\;g_{MeOH}/g_{PVAc}$ and $h_{gap}=200\;\mu m$ ), drying at $T_{substrate}=20\;{}^{\circ}C$ and otherwise ambient conditions, measured with 3D-µPTV and evaluated with mean refractive index. The flow field clearly shows convective instabilities. (**a**) The 3D tracer-particle trajectories. (**b**) Transient vertical tracer particle positions. The convective instability stops during drying at $t_{crit,Ma}=144\;s$. The extracted drying curves are additionally given for an evaluation with the refractive index of the coating solution $n_{0}$ and the dry polymer $n_{dry}$, respectively (black curves).

Keeping the coating gap fixed, but increasing the initial solvent load of the coating solution to $X_{0}=1.5\;g_{MeOH}/g_{PVAc}$, changes the flow field drastically. The 3D trajectories given in Figure 4a show vertical as well as lateral flow within the drying film. The transient vertical tracer positions in Figure 4b show that in the first ≈2 min, there is significant upward and downward flow simultaneously. The film is convectively unstable. When drying progresses, the vertical flow decelerates until, at a critical drying time $t_{crit,Ma}=144\;s$ (black dotted vertical line), the last upward-moving tracer particle reaches its highest position. Following this, the instability ceases and the flow field is dominated by film shrinkage only.

The flow fields reported in Figure 3 and Figure 4 are a good representation of all conducted drying experiments measured with 3D-µPTV. All films with an initial solvent load of $X_{0}=1\;g_{MeOH}/g_{PVAc}$ ($h_{gap}=100$ and 200 μm) were stable. Experiments with $X_{0}=1.5\;g_{MeOH}/g_{PVAc}$ and $h_{gap}=100$ to 200 μm as well as with $X_{0}=2\;g_{MeOH}/g_{PVAc}$ and $h_{gap}=50$ to 200 μm were initially unstable with varying critical drying times $t_{crit,Ma}$, denoting the transition to convectively stable. Three repetitions of a drying experiment with $X_{0}=1.5\;g_{MeOH}/g_{PVAc}$ and $h_{gap}=50\;\mu m$ show a mixed picture with one experiment being initially stable and two others being unstable with $t_{crit,Ma}<20\;s$. This indicates that the initial conditions for these experiments are very close to a stability threshold. All critical drying times will be further discussed in Section 3.5.

### 3.2. Film Thickness

**Figure 5:**
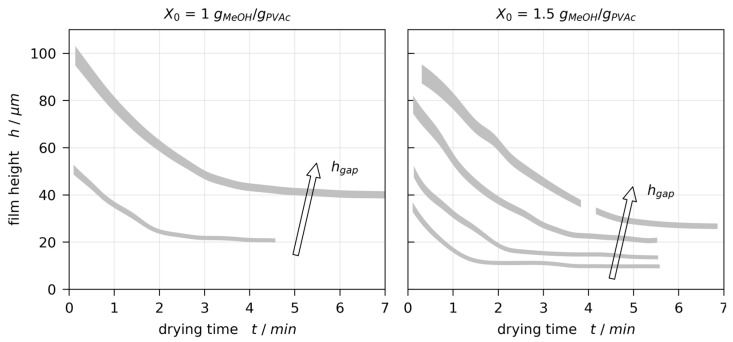
Comparison of drying curves for poly(vinyl acetate)-methanol films at $T_{substrate}=20\;{}^{\circ}C$ and ambient drying conditions. The filled areas denote experimental results from 3D-µPTV measurements accounting for refractive index uncertainty in the evaluation. The true drying curves would align with the lower bounds of the grey areas at t=0 s and with the upper bounds for solvent depleted films. (**a**) $X_{0}=1\;g_{MeOH}/g_{PVAc}$, $h_{gap}=100$ and 200 μm. (**b**)$\;X_{0}=1.5\;g_{MeOH}/g_{PVAc}$, $h_{gap}=50$, 100, 150, and 200 μm.

The drying curves (transient film thickness) extracted from 3D-µPTV results are given in Figure 5. The true drying curve would align with the lower bounds of the grey areas at t=0 s and the upper bounds when completely dry due to the refractive index dependent experiment evaluation. In order to validate the film height measured with 3D-µPTV, additional poly(vinyl acetate)-methanol films ($X_{0}=1$ and $2\;g_{MeOH}/g_{PVAc}$, $h_{gap}=200\;\mu m$) have been coated on a glass substrate with temperature-controlled support and dried for at least two hours. The dry film thickness was measured using 3D-µPTV as well as a physical measuring probe (ID-H, 543-561D, Mitutoyo, Kawasaki, Japan) at three points in each film. Results comparing the two independent measurement techniques are given in Table 1. They show excellent agreement, validating the film thickness derived from 3D-µPTV for solvent depleted films. The experimentally derived drying curves (Figure 5) follow the typical behavior during film drying, with an initial constant rate period (constant slope) where the film drying is governed by solvent evaporation, followed by a falling rate period (plateau) where the solvent diffusion in the film limits the solvent evaporation.

The glass transition temperature of pure poly(vinyl acetate), measured with differential scanning calorimetry, was reported to be $T_{g}\approx 29\;{}^{\circ}C$, whereas a small addition of methanol ($X=0.046\;g_{MeOH}/g_{PVAc}$) reduced the glass temperature to $T_{g}\approx 15\;{}^{\circ}C$ [19]. With a drying temperature of T≈20 °C used in this work, the data imply that glass transition during drying may occur only at very late stages of drying. In addition, the strong concentration-dependency of the diffusion coefficient was reported to occur below $X\approx 0.3\;g_{MeOH}/g_{PVAc}$ [53,57], indicating that the end of the constant rate period is likely to occur well before $T_{g}$ exceeds the drying temperature. We have previously reported transient vertical concentration profiles in drying poly(vinyl acetate)-methanol films measured with Raman spectroscopy at drying temperatures of 20 °C and 40 °C and an initial solvent load comparable to those employed in this work [19,47,53,57]. The data were in excellent agreement with a one-dimensional film drying model, accounting for Fickian diffusion and a concentration-dependent diffusion coefficient in the film, despite not explicitly accounting for the effect of glass transition at drying temperatures of 20 °C. In light of these findings, we are certain that glass transition is not the reason for the reported convective instability to stop but may occur only well after $t_{crit,Ma}$. However, this may be a relevant factor in other polymer systems (e.g., PMMA). A quantitative assessment of this phenomenon will be addressed in future work.

### 3.3. Velocity Distributions

**Figure 6:**
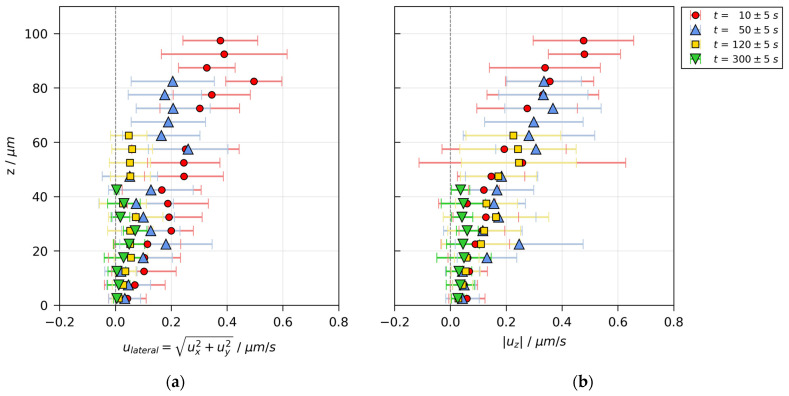
Velocity distribution in a poly(vinyl acetate)-methanol film ($X_{0}=1\;g_{MeOH}/g_{PVAc}$ and $h_{gap}=200\;\mu m$ ), drying at $T_{substrate}=20\;{}^{\circ}C$ and otherwise ambient conditions, measured with 3D-µPTV and evaluated with mean refractive index. Data are averaged laterally and in vertical slices of Δz=5 μm. The flow field is dominated by film shrinkage. (**a**) Lateral velocity over film height. (**b**) Absolute vertical velocity over film height.

Additional information on the flow field can be extracted by calculating the transient velocity distributions. Figure 6 shows the lateral and vertical velocity magnitude over the film height in the initially stable film (Figure 3) at several drying times. The data were averaged laterally and in vertical slices of Δz=5 μm. It can be seen that the magnitude of the vertical velocity (Figure 6b) is highest at the film surface and monotonically decreases towards the bottom of the film (red and blue markers), which is to be expected for flow fields governed by film shrinkage during the constant rate period. At a later stage during drying after the constant rate period (green markers), the magnitude of vertical velocity is very low (<0.1 μm/s) and almost constant over the film height. The lateral velocity, depicted in Figure 6a, is of similar magnitude as the vertical velocity in the early stage of drying (red markers), but decelerates faster in later stages of drying (blue and yellow markers).

**Figure 7:**
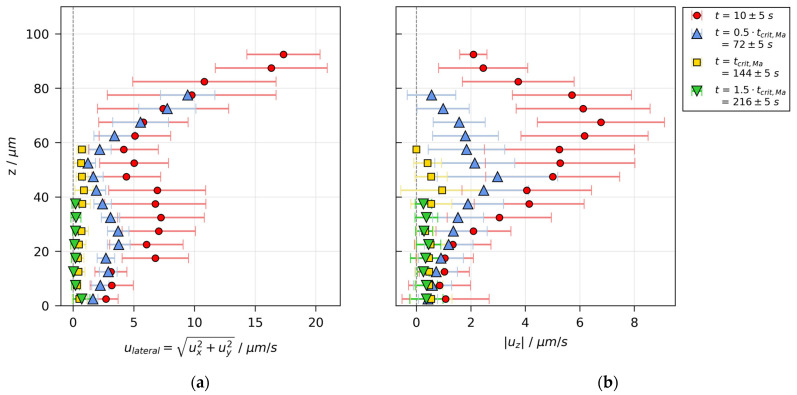
Velocity distribution in a poly(vinyl acetate)-methanol film ($X_{0}=1.5\;g_{MeOH}/g_{PVAc}$ and $h_{gap}=200\;\mu m$ ), drying at $T_{substrate}=20\;{}^{\circ}C$ and otherwise ambient conditions, measured with 3D-µPTV and evaluated with mean refractive index. Data are averaged laterally and in vertical slices of Δz=5 μm. The flow field shows convective instabilities until $t_{crit,Ma}=144\;s$. (**a**) Lateral velocity over film height. (**b**) Absolute vertical velocity over film height.

The velocity distribution of the initially unstable film (Figure 4) is shown in Figure 7. In contrast to the velocity distribution for a stable film, the lateral velocity magnitude is significantly larger than the vertical one. While convectively unstable, the lateral velocity magnitude, depicted in Figure 7a, is given by the red markers for t=10±5 s and blue markers for $t=0.5\cdot t_{crit,Ma}=72\pm 5\;s$. It can be seen that the velocity is highest at the surface, decreasing to a local minimum at about two-thirds of the film height and showing a local maximum at about one-third of the film height. On the other hand, the vertical velocity magnitude, shown in Figure 7b, shows only one maximum at approximately two-thirds of the film height, where the lateral velocity is minimal. During the course of drying, the overall velocity magnitude, lateral as well as vertical, decreases noticeably from up to ≈17 μm/s and ≈7 μm/s, respectively, until at the threshold from convectively unstable to stable ($t_{crit}$, yellow markers) the velocity has reduced to less than 1 μm/s. The observation that the highest overall velocity magnitude occurs laterally at the surface of the film, despite the fact that a drying film has its lowest solvent concentration and therefore the highest viscosity at the surface, is a clear indicator for Marangoni flow as the driving force of the convective instability. This attribution is also reflected in the upper limit of the Bond number. It was calculated using the pure PVAc density and the highest measured film thickness $h_{0,max}\approx 100\;\mu m$ in the numerator, as well as the lowest possible surface tension (pure MeOH) in the denominator. The resulting upper limit is $Bo_{max}=5\cdot 10^{-3}\ll 1$, which underlines the negligibility of buoyancy compared to Marangoni instabilities. Due to the surface tension increase and height decrease during drying, realistic values are likely to be even smaller.

The velocity distribution indicates that the instability has the form of vertical convection cells with a driving lateral flow at the surface, vertical flow in the upper third of the film, and a lateral backflow in the lower third of the film. Consequently, it can be concluded that the observed convective instabilities are short-scale Bénard–Marangoni convection cells.

### 3.4. Convection Cell Pattern and Size

**Figure 8:**
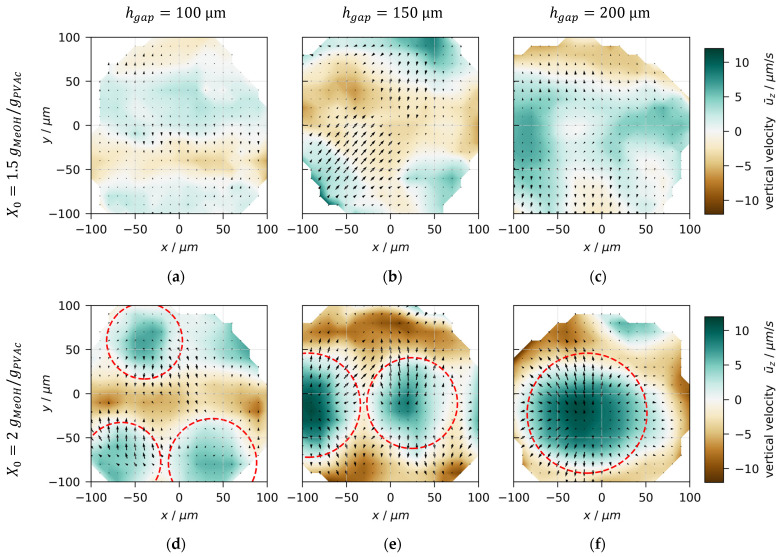
Top-view of flow field in poly(vinyl acetate)-methanol films, drying at $T_{substrate}=20\;{}^{\circ}C$ and otherwise ambient conditions. Data at t=10 s drying time. The colored background shows the height-averaged vertical velocity and the black arrows indicate the lateral velocity at the film surface. The discrete tracer data were interpolated on a regular grid for better visualization. (**a**–**c**) $X_{0}=1.5\;g_{MeOH}/g_{PVAc}$. (**d**–**f**) $X_{0}=2\;g_{MeOH}/g_{PVAc}$. The red circles were fitted to white areas, denoting regions of zero height-averaged vertical velocity, in order to extract lateral cell sizes. (**a**,**d**) $h_{gap}=100\;\mu m$. (**b**,**e**) $h_{gap}=150\;\mu m$. (**c**,**f**) $h_{gap}=200\;\mu m$.

To shed further light on the form of the convection cells, Figure 8 depicts the top view of multiple initially unstable films with initial solvent loads $X_{0}=1.5$ and $2\;g_{MeOH}/g_{PVAc}$ (rows) as well as coating gaps $h_{gap}=100$, 150, and 200 μm (columns) at t=10 s drying time. The colored background shows the height averaged vertical velocities, with areas in green indicating an upward flow and areas in brown a downward flow. In addition, the black arrows represent the lateral surface flow. For all experiments, the lateral surface flow is directed away from areas with upward flow (green) towards areas with downward flow (brown). In addition, there is a trend that for increasing coating gap (left to right) the lateral as well as vertical velocity magnitude increases.

The lateral shape of convection cells appears to be irregular for initial solvent loads of $X_{0}=1.5\;g_{MeOH}/g_{PVAc}$ (first row) but is close to circular for $X_{0}=2\;g_{MeOH}/g_{PVAc}$ films (second row). The red circles in Figure 8d–f were matched to the white areas surrounding the cell centers, which indicate regions with zero height-averaged vertical velocity. We use the diameter of the matched red circles as an indicator for the lateral convection cell size. This differs from approaches in the literature, where either center-to-center or edge-to-edge distances are commonly used, which are averaged over a large lateral field-of-view. It has to be noted that due to the limited lateral observation area of 3D-µPTV, the observed cell sizes are not necessarily representative of the entire film.

**Figure 9:**
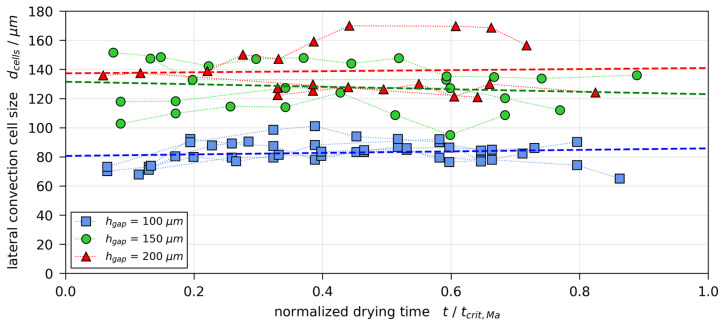
Transient lateral convection cell size from films with an initial solvent load of $X_{0}=2\;g_{MeOH}/g_{PVAc}$ and different initial film heights ($h_{gap}=100$, 150, and 200 μm ). Each set of markers indicate experimental results of a single cell (see Figure 8, red circles), while the dashed lines are the respective linear fits.

The data on lateral cell size that we were able to extract from films with $X_{0}=2\;g_{MeOH}/g_{PVAc}$ are plotted in Figure 9, over the normalized drying time $t/t_{crit,Ma}$. It can be seen that there is a trend for the cell size to increase with initial film height (increasing $h_{gap}$) and that the lateral cell size remains fairly constant during drying. These findings are in good agreement with results from Bassou and Rharbi, who reported a linear dependency of lateral cell size to film thickness and a constant cell size over 75% of drying time in drying films of polystyrene-toluene at 22.5 °C [18].

### 3.5. Stability Threshold and Upper Limit of Marangoni Numbers

**Figure 10:**
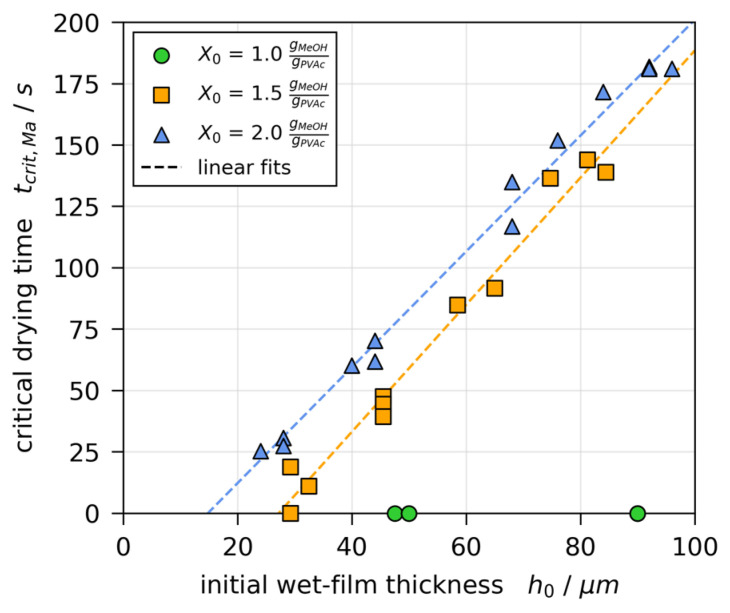
Critical drying times $t_{crit,Ma}$, denoting the transition from convectively unstable to stable during 3D-µPTV drying experiments, plotted over initial wet film thickness $h_{0}$ from Figure 5.

Figure 10 shows the critical drying times $t_{crit,Ma}$, at which initially convectively unstable films become stable, over the initial wet-film thickness $h_{0}$ for all drying experiments. While all films with $X_{0}=1\;g_{MeOH}/g_{PVAc}$ were initially stable ($t_{crit,Ma}=0\;s$, green markers), the critical drying time decreases linearly with decreasing $h_{0}$ for $X_{0}=1.5$ and $2\;g_{MeOH}/g_{PVAc}$ (orange and blue markers), respectively. The linear fits (dashed lines) intersect with the *x*-axis at $h\left(X_{0}=1.5\right)=27.1\;\mu m$ and $h\left(X_{0}=2\right)=14.8\;\mu m$, respectively. This implies that there is a critical film thickness, varying with $X_{0}$, below which films stay convectively stable. This is in qualitative agreement with theoretic findings by de Gennes et al. [15]. Furthermore, it is in line with the definition of the Marangoni numbers, which implies that a viscosity increase ($X_{0}$ decrease) and a film thickness decrease results in smaller values of the Marangoni numbers and therefore act stabilizing. For the rigorous quantitative assessment of the Marangoni numbers at the onset of drying, the vertical surface tension difference Δσ is required. Assessing the stop of convective instabilities requires the additional knowledge of transient solution properties, resulting in transient Marangoni numbers, likely to decrease during drying, because of the film shrinkage and strong viscosity increase. Therefore, it is likely that a critical value of the thermal or solutal Marangoni number is undercut during drying, hence the stop of the convective instabilities.

In order to assess the upper limit of thermal and solutal Marangoni numbers for the reported film drying experiments, we made assumptions of the vertical temperature and concentration gradient based on previously reported drying experiments of the same polymer solution [19,42]. Additional details are given in Appendix B. It was found that the upper limit of thermal and solutal Marangoni numbers are $Ma_{T,max}=6$ and $Ma_{s,max}=11,429$, respectively. It has to be noted that these are only very coarse estimates and that realistic vertical gradients combined with the strong viscosity increase during drying, would likely result in significantly lower Marangoni numbers. However, a more detailed analysis requires the knowledge of the transient vertical surface tension difference and all solution properties in Equations (3) and (4). This will be addressed in the near future. Nevertheless, the coarse assessment of the upper limits reveals that the thermal Marangoni number is significantly smaller than reported values of the critical thermal Marangoni number found in literature. In addition, the assessed upper limit of the solutal Marangoni number is orders of magnitude larger than the thermal one. This implies that for the reported film drying experiments, solutally induced Marangoni convection is the dominant effect causing the observed convection cells. Several authors came to the same conclusion for different material systems [18].

## 4. Conclusions

Results from drying experiments under ambient conditions with poly(vinyl acetate)-methanol thin films, investigated with three-dimensional micro particle tracking velocimetry (3D-µPTV), have been presented. It was found that films with an initial solvent load of $X_{0}=1\;g_{MeOH}/g_{PVAc}$ and an initial wet film thickness of $h_{0}\leq 100\;\mu m$ are convectively stable during the entire course of drying, whereas films with $X_{0}=1.5$ and $2\;g_{MeOH}/g_{PVAc}$ initially exhibit short-scale Bénard–Marangoni convection cells and become convectively stable during the constant rate period. The lateral convection cell size was shown to decrease with decreasing initial film thickness but stays fairly constant during the course of drying, which is in good agreement with experimental data from Bassou and Rharbi [18]. An initial assessment of the upper limit of thermal and solutal Marangoni numbers indicates that the solutal effect is the main driving force of the observed convective instabilities.

## Figures and Tables

**Table 1:** Comparison of dry-film thicknesses measured using 3D-µPTV and physical probe.

$X_{0}/\left(g_{MeOH}/g_{PVAc}\right)$	$h_{gap}/\mu m$	$h_{dry,\mu PTV}/\mu m$	$h_{dry,probe}/\mu m$	$\Delta h_{dry}/\mu m$
1	200	40.2±2.0	39.5±1.7	0.7±3.7
2	200	23.4±1.6	22.4±1.4	1.0±3.0

## Data Availability

The data presented in this study are available in the article.

## Appendix A

Several material properties were used for 3D-µPTV drying experiment evaluation as well as an initial assessment of the upper limit of Marangoni numbers. The properties for pure methanol and pure PVAc are given in Table A1 and Table A2, respectively. Several properties of the mixture have been calculated using ideal mixing rules given in Table A3. The concentration-dependent surface tension and mutual mass diffusion coefficient are provided in Table A4.

**Table A1:** Material properties of pure liquid methanol.

Property	Unit	Value	Temperature Range/°C	Source
ρ	$\mathrm{kg}/m^{3}$	791.5	20	own data (DMA 4100 M, Anton Paar)
n	−	1.3295	20	own data (Abbemat, Dr. Kernchen)
λ	W/(m⋅K)	0.20	10 to 30	[73]
$c_{p}$	J/(kg⋅K)	2508	20	[73]

**Table A2:** Material properties of pure poly(vinyl acetate).

Property	Unit	Value	Temperature Range/°C	Source
ρ	$\mathrm{kg}/m^{3}$	1185.1	20	[74]
n	−	1.4672	20	own data (Abbemat, Dr. Kernchen)
λ	W/(m⋅K)	0.159	≈20	[75]
$c_{p}$	J/(kg⋅K)	1449	20	[75]

**Table A3:** Ideal mixing rules with $x_{i}$ and $\varphi_{i}$ denoting mass fraction and volume fraction of component i, respectively.

Property	Mixing Rule Equation	Source
ρ	${\left(\underset{i}{\sum }x_{i}\times \rho_{i}^{-1}\right)}^{-1}$	[73]
n	$\underset{i}{\sum }\varphi_{i}\times n_{i}$	[19]
λ	$\underset{i}{\sum }\underset{j}{\sum }\frac{2\times \varphi_{i}\times \varphi_{j}}{\lambda_{i}^{-1}+\lambda_{j}^{-1}}$	[73]
$c_{p}$	$\underset{i}{\sum }x_{i}\times c_{p}$	[73]

**Table A4:** Concentration-dependent surface tension σ and mutual mass diffusion coefficient D from pol(vinyl acetate)-methanol solutions with ϑ being the temperature in °C, as well as $x_{MeOH}$ and $X_{MeOH}$ being the methanol mass fraction and methanol solvent load, respectively.

Equation	Parameters	T-Range/°C	Source
$\sigma =A\times exp\left\{0.72\times \left(x_{MeOH}+B\right)\right\}-C\times x_{MeOH}$	A=0.0949×ϑ+15.858 B=0.005×ϑ+1.2982 C=−0.2032×ϑ+60.111	25 to 40	[42]
$D_{MeOH,PVAc}=exp\left(-\frac{A+B\times X_{MeOH}}{1+C\times X_{MeOH}}\right)$	A=30.39 B=111.17 C=5.57	20	[47]

## Appendix B

In order to assess an upper limit of the thermal and solutal Marangoni numbers (Equations (3) and (4)), the highest measured initial wet-film thickness was taken $h_{max}\approx 100\;\mu m$ and the lowest initial viscosity $\eta_{min}=\eta \left(X_{0}=2\;g_{MeOH}/g_{PVAc}\right)=0.15\;\mathrm{Pa}\cdot s$. The vertical surface tension difference due to a vertical temperature gradient was calculated using material properties from [42] and a worst-case assumption of $\Delta T_{vertical}=-10\;{}^{\circ}C$ based on our findings from previously reported drying experiments with deliberately inhomogeneous drying conditions over a Teflon substrate inlay, described in the introduction [42]. This results in a thermally induced vertical surface tension difference of $\Delta \sigma {\left(T\right)}_{max}=0.9\;\mathrm{mN}/m$. The lowest value for the thermal diffusivity is $a_{min}=a_{MeOH}=\lambda /\left(\rho \cdot c_{p}\right)=1.0\cdot 10^{-7}\;m^{2}/s$. Entering these material properties in Equation (3) gives an upper limit for the thermal Marangoni number of $Ma_{T,max}=6$.

It has to be noted that worst-case assumption of $\Delta T_{vertical}=-10\;{}^{\circ}C$ is very unlikely to occur in films subject to evaporative cooling and a thickness in the order of 10 to 100 μm, due to thermal conduction of the film itself. A common approach to assess whether the heat transfer in the gas phase or the heat conductivity in the film is limiting the resulting heat flux, is to consult the dimensionless Biot number $Bi=\alpha_{gas}h/\lambda_{film}$ [73]. A very large Biot number Bi≫1 implies that the heat flux is limited by the thermal conductivity of the film. In such a case, a common simplification is to assume a constant and uniform temperature of the gas phase, while strong temperature gradients in the film may arise. The complementary case Bi≪1 implies that the heat flux is limited by the heat transfer in the gas phase. In this case, a common simplification is to assume a uniform film temperature [73]. Considering the experiments presented in this work, the mild drying conditions (ambient drying) result in small values of the gas phase heat-transfer coefficient $\alpha_{gas}$, which is likely to be in the order of 1 to $10\;W/\left(m^{2}K\right)$. Using the larger limit in combination with $h_{max}\approx 100\;\mu m$ and the lowest possible value of the film’s heat conductivity (pure PVAc) $\lambda_{film}\geq 0.159\;W/\left(m\cdot K\right)$, the resulting upper limit of the Biot number would be $Bi_{max}=6\cdot 10^{-3}\ll 1$. Even for extremely fast drying ($\alpha_{gas}\approx 200\;W/\left(m^{2}K\right)$), the Biot number would still be Bi=0.13<1. Hence, it is rather unlikely that large vertical temperature differences arise in films with a thickness in the order of 10 to 100 μm purely induced by evaporative cooling. This could only be achieved in significantly thicker films or with additional active cooling of the film’s free surface (e.g., forced convective airflow with low temperature) and simultaneous heating of the bottom of the film.

The maximum of the vertical surface tension difference due to a vertical solvent gradient was found by combining experimental concentration profiles reported in [19] with the surface tension data in [42]. This gives a solutally induced vertical surface tension difference of $\Delta \sigma {\left(x\right)}_{max}=2.4\;\mathrm{mN}/m$. The diffusion coefficient decreases by several orders of magnitude with decreasing solvent content [47]. Solvent loads of X=0, 0.5, and $2\;g_{MeOH}/g_{PVAc}$ correspond to a mutual diffusion coefficients of $D=6.3\cdot 10^{-14}$, $1.4\cdot 10^{-10}$, and $9.1\cdot 10^{-10}\;m^{2}/s$, respectively. Taking the lowest value for the limiting case of pure polymer would result in an upper limit for the solutal Marangoni number (Equation (4)) of $2.5\cdot 10^{7}$. The pure polymer, however, is solidified and therefore no convective instability may occur. A more realistic worst-case assumption is to take half of the lowest initial solvent load from experiments $X=0.5\;g_{MeOH}/g_{PVAc}$ for the calculation of the diffusion coefficient $D_{min}=D\left(X=0.5\;g_{MeOH}/g_{PVAc}\right)=1.4\cdot 10^{-10}\;m^{2}/s$. This results in an upper limit for the solutal Marangoni number of $Ma_{s,max}=11,429$.

## Appendix C

Uncertainties of the presented results are likely to occur due to the experimental drying conditions or the complex reconstruction of particle positions based on the observed diffraction rings, presented in [70]. Since the drying experiments were conducted without controlled airflow in the gas phase above the film, the drying rate may slightly vary between individual experiments due to naturally occurring fluctuations and methanol vapor accumulation in the gas phase above the film during drying. This is reflected by the mild scattering of $t_{crit,Ma}$ presented in Figure 10.

Regarding the µPTV evaluation, it was found in the original publication that the used ring-detection algorithm has an accuracy of sub-pixel resolution [69]. The main error occurring during ring detection is that rings are not detected altogether when too many tracer particles result in many laterally overlapping diffraction rings [69]. As this results only in a reduced amount of detections, it does not affect the quality of the presented data. Regarding the found lateral particle positions and velocity distributions, it is possible to calculate the uncertainty by assuming a small error of 3 px in the detected ring positions and calculate the error propagation based on the conversion steps presented in [70]. Considering only a single particle this results in a worst-case error of the lateral position $\Delta {\left\{x,y\right\}}_{single}\leq 1.6\;\mu m$ and $\Delta u_{lateral,single}\leq 1.6\;\mu m/s$. Assuming a statistical error distribution, the error significantly decreases due to trajectory smoothing using a Savitzky–Golay filter and additional averaging over multiple trajectories, which has been discussed in detail in [70]. The residual error after smoothing and averaging is $\Delta {\left\{x,y\right\}}_{av}\leq 0.2\;\mu m$ and $\Delta u_{lateral,av}\leq 0.2\;\mu m/s$.

Regarding the reconstruction of the vertical particle positions and velocities, a rigorous error propagation calculation is unfeasible due to the complex reconstruction and calibration procedure presented in [70]. The impact of the sample refractive index on vertical particle position reconstruction was already discussed in the main text and the error due to reconstruction can be deduced from the experimental validation of the dry film thickness presented in Table 1 $\Delta z\approx \Delta h_{dry}/h_{dry}$. The resulting worst-case error would be $\Delta z_{max}\leq 19\%$, whereas the averaged values would imply $\Delta z_{av}\leq 5\%$. Since the vertical velocities were calculated from the vertical positions, the same limits apply to $\Delta u_{z}$.
